# Stimuli Influencing Engagement, Satisfaction, and Intention to Use Telemedicine Services: An Integrative Model

**DOI:** 10.3390/healthcare10071327

**Published:** 2022-07-18

**Authors:** Ruhul Amin, Md. Alamgir Hossain, Md. Minhaj Uddin, Mohammad Toriqul Islam Jony, Minho Kim

**Affiliations:** 1 Department of Management, Jashore University of Science and Technology, Jashore 7408, Bangladesh; ruhulbsmrstu@gmail.com; 2 Department of Management, Hajee Mohammad Danesh Science and Technology University, Dinajpur 5200, Bangladesh; shamimru@gmail.com; 3 Department of Accounting and Information Systems, Jatiya Kabi Kazi Nazrul Islam University, Mymensingh 2220, Bangladesh; minhaj0172@gmail.com; 4 Department of Management, Jatiya Kabi Kazi Nazrul Islam University, Mymensingh 2220, Bangladesh; jony.jkkniu@gmail.com; 5 Department of International Trade, Jeonbuk National University, Jeonju 54896, Korea

**Keywords:** telemedicine, stimulus-organism-response framework, engagement, satisfaction, continuous usage intention

## Abstract

Telemedicine ensures quality, cost-effective, and equally accessible healthcare services for everyone. Nonetheless, a poor usage rate could curb its progression in developing cultures like Bangladesh. Therefore, this research examines how external stimuli promote the continuous usage intentions of synchronous telemedicine services through engagement and satisfaction by deploying the stimulus-organism-response framework. A final sample of 312 telemedicine users was analyzed using the structural equation modeling in AMOS. The average age of the participants was 26.28 (std. deviation 5.53), and their average use of telemedicine was 2.39 times (std. deviation 1.31) over the last six months. This study empirically endorsed that the stimuli, including performance expectancy, information quality, and contamination avoidance, as well as organismic factors such as engagement and satisfaction, directly impacted the continuance desires for telemedicine use. In addition, the analyses validated the mediation roles of engagement and satisfaction. Furthermore, performance and effort expectancies influenced engagement, which affected satisfaction along with performance expectancy, functionality, and information quality. Accordingly, telemedicine facilitators should integrate these critical attributes into the system to sustain engagement, satisfaction, and usage intentions. This study has pioneered the effects of performance and effort expectancies on continuous usage intentions facilitated by engagement and satisfaction in the telemedicine landscape.

## 1. Introduction

Telemedicine is a form of electronic health fabricated on computers, smartphones, and wireless sensors and providing healthcare services to remote patients [1]. Telemedicine has three forms: synchronous vs. asynchronous, data transfer and storage, and automating and robotic telemedicine services [2]. The current study focuses on synchronous telemedicine, through which patients can take up live consultations with doctors using online mediums like web portals or mobile applications [3]. The global real-time telemedicine market will approach USD 23,406.05 million in 2022 and 33,575.48 million by 2026 [3]. However, the market volume of synchronous telemedicine varies from region to region; for instance, in Asia, the market size is expected to reach USD 10,833.75 million in 2022 and 17,254.54 million by 2026 [4], where China and Bangladesh will generate USD 7833.61 million and 26.49 million, respectively, in 2022 [4,5]. The growing trend of this disruptive technology is triggered by the increasing usage of computers, smartphones, social networks, web portals, mobile applications, and internet of things (IoT) devices [6]. Additionally, the COVID-19 pandemic has boosted the demand for telemedicine globally [7,8], and Bangladesh is not an exception. Bangladesh is a highly populated country with more than 160 million people, and most of them live in rural areas. But there are only 96,000 registered doctors, most of whom serve in urban areas [9]. Therefore, such research in Bangladesh is urgent to bring marginal patients under the umbrella of quality healthcare services.

Telemedicine saves time and transportation costs for distant patients [10]. It lets them access quality healthcare services at a fair price [11], thus setting up parity of medical aid between rural and urban people. In particular, Artificial Intelligence (AI)-based telemedicine applications improve autistic people’s quality of life by developing their oral, social, and non-verbal interaction skills [12]. In addition, working parents with childcare responsibilities might find telemedicine more convenient than in-person visits because they can easily schedule online visits around their busy times. Similarly, telemedicine improves the efficiency of medical professionals like physicians, consultants, and nurses by allowing them to manage more remote patients within a limited time and cost [13]. Remarkably, the governments and medical service providers have taken telemedicine as an effective solution to crises led by catastrophes like COVID-19 [10], severe drought, and bushfires [14]. Though the benefits of telemedicine are extensively recognized, there are many obstructions to engaging users with it. For instance, poor functional quality and data security, lack of infrastructural support, and inability to perform in-person medical tests were acknowledged as the major impediments to telemedicine services [15]. Moreover, Lunney et al. [7] revealed that lack of patient training on telemedicine platforms, lack of facilitating conditions, and inappropriate remuneration given to the physicians for delivering telemedicine services are the main barriers to making telemedicine feasible. Poor internet connectivity and lack of computer literacy make telemedicine services more difficult, particularly for elderly patients [8].

Therefore, telemedicine facilitators are concerned not only with the challenges of how they can deploy telemedicine services and increase user engagement but also with whether the engagement can subsequently produce higher satisfaction and usage intentions. Engagement, defined as an individual’s cognitive, affective, and behavioral exposure to their work [16], has been documented as a critical variable in the technology literature because it results in its successful adoption [17]. Healthcare institutions mainly use engagement for formulating policy, which is then implemented to develop a new care model, improving service quality and health outcomes of patients [18]. Nonetheless, research gaps between engagement and telemedicine usage intentions are manifold. Firstly, several studies [19,20] have pointed out how the stimuli of performance expectancy and effort expectancy generate greater engagement among technology users. Stimuli are defined as the band of external environment factors that provoke an individual’s emotional and behavioral changes [21]. However, the relationship of these stimuli with engagement remains inconclusive in the telemedicine landscape. Secondly, engagement might also develop functional outcomes, like satisfaction and long-term usage intentions, as found in the digital healthcare literature. For example, a study by Wang et al. [22] argued that healthcare professionals using AI technologies like Chabot and robots express satisfaction and continuous usage intentions (CUI). In contrast, such research in the context of video-based teleconsultations run on mobile apps or web portals remains untested. Thirdly, earlier research on hotel information management systems (HIMS), mobile-business intelligence (m-BI), and gaming apps presents engagement as a mechanism through which stimuli can sustain usage intentions [23,24]. However, such a strong mediation effect remains unexamined in the telemedicine context. We conduct a mediation test because it assists in comprehending the complex relationships between study variables.

Satisfaction, an individual’s emotional response to the stimuli [21], also enhances consumers’ post-adoption behaviors, including the CUI, word-of-mouth, repeat purchase, or loyalty toward any technology field [25]. Consequently, some researchers [25,26] concluded that telemedicine stimuli, such as performance expectancy, effort expectancy, facilitating conditions, functionality, and information quality significantly impact user satisfaction. Still, very few studies have evinced how these external stimuli can improve post-adoption behaviors toward telemedicine via satisfaction. For example, to the best of our knowledge, only Zhou et al. [27] reported how effort expectancy and information quality affect usage intentions of telemedicine through satisfaction. However, other essential stimuli like performance expectancy and facilitating conditions might generate usage intentions via satisfaction, which is unexplored in telemedicine. The current study considers performance expectancy and facilitating conditions because both play significant roles in technology adoption [28]. Another important variable that we incorporate into our model is contamination avoidance, as it has gained the attention of researchers due to COVID-19. For example, Herget and Krey [29] showed how contamination avoidance contributes to performance expectancy and usage intention of mobile technology. Nevertheless, we did not find any study exploring contamination avoidance’s influence on the CUI of telemedicine.

The present research intends to address these needs by enumerating how the external stimuli of telemedicine can heighten the CUI. More importantly, we dissect the mediating mechanisms, such as engagement and satisfaction, through which stimuli can achieve this. We establish the current research model by using stimulus-organism-response (S-O-R) theory [21], wherein we adjoin the Unified Theory of Acceptance and Use of Technology (UTAUT2) model [30], the expectation confirmation model (ECM) [31], and the Adapted-Mobile App Rating Scale (A-MARS) [32]. Many researchers have empirically validated their findings by applying these theoretical lenses but have not integrated these approaches in the telemedicine context. We combine the contamination avoidance, information quality, and functionality variables with the UTAUT2 constructs, which is rare in the literature. Therefore, we believe this holistic model will help the concerned stakeholders, such as telemedicine providers, policymakers, promoters, managers, and the federal authorities, understand users’ stimuli, attitudes, and behavior toward telemedicine from a broader perspective and provide inputs for formulating and implementing the blueprints needed to promote and sustain telemedicine, particularly in a developing context like Bangladesh.

In line with the abovementioned gaps, this study addresses the research questions: *RQ1:* How do stimuli influence the CUI of telemedicine services? *RQ2:* Do engagement and satisfaction mediate the impacts of stimuli (i.e., performance expectancy, facilitating condition, and effort expectancy) on the CUI of telemedicine services? *RQ3:* What are the antecedents to building engagement and satisfaction?

The subsequent sections of this paper are as follows. Section 2 describes the theoretical bases of past research reviews and develops the research model. Section 3 develops the research hypotheses, followed by the research methodology and empirical results in Section 4 and Section 5, respectively. Section 6 includes discussions on key findings. Section 7 states the theoretical and practical contributions. Section 8 concludes the paper with limitations and guidelines for future research.

## 2. Theoretical Grounds and Literature Review

### The S-O-R Framework in Technology and Telemedicine

The S-O-R framework, introduced by Mehrabian and Russel in 1974, postulates that the stimuli, which are external to an individual (the “stimulus”), drive them to reveal internally felt emotional reactions (the “organism”) to the environment. Subsequently, this impulsive experience forms their behavioral patterns (the “response”) [21]. So, the stimulus refers to the set of external environment inputs immediately causing an individual’s emotional and behavioral changes. The second component of the S-O-R theory is the organism, which includes an individual’s cognitive and affective states that act as the intermediary mechanisms between the stimulus and response variables [21]. The cognitive state mainly denotes an individual’s beliefs or thoughts, by which they process the information of a phenomenon experienced in their surroundings. The affective state refers to the emotional responses (e.g., satisfaction and pleasure) to the stimuli [21]. Finally, the response factor consists of the behavioral outcomes, such as approach and avoidance, which are instantly derived from the organismic components or directly from the stimuli [21]. More specifically, approach behaviors reflect an individual’s positive reactions, whereas avoidance behaviors come from adverse responses [33].

Several researchers have identified some stimuli in the information systems (IS) domain by applying the S-O-R paradigm. In the context of mobile gaming, for instance, Hsiao and Tang [34] displayed that critical mass, content timeliness, social interaction, and media richness significantly evoke the players’ inner experiences, such as attachment and conformity, which ultimately result in their behavioral intentions to play the game. In online learning, interactivity, sociability, and media richness were shown as the significant environmental factors, illuminating learners’ experiences of telepresence and social presence followed by their continuous intentions of using Massive Open Online Courses (MOOCs) [33]. Choi [35], a social commerce researcher, reported in an article that perceived usefulness, ease of use, joy, and pride significantly increase the social commerce users’ intentions. A similar study in the context of branded festival apps concluded that perceived usefulness, aesthetics, and gamification could rouse the festival goers’ emotional responses and engagement, which in turn could improve their brand loyalty [36].

However, few studies applied the S-O-R framework to explain the context of telemedicine. For example, Sreejesh et al. [37], in their online healthcare research, demonstrated the technology-enabled co-creation as an incentive option for the users of online medical care consultancy to express their feelings of co-presence, spatial presence, service experience, and positive value perception, which led to patients’ frequent usage behavior. In a similar domain, another study by Chang et al. [38] argued that justice perceptions towards online doctor consultations boost the patients’ trust and satisfaction levels. The aftermath of these organismic feelings is the sustained intention to use teleconsultation services [38]. In mobile health (mHealth) research, Shan et al. [39] presented the physician- and patient-generated information as antecedent variables that improve the patients’ trust in mHealth services. Afterward, trust increases their online choice of doctors [39]. A recent study by Kumar et al. [40] proposed certain agent-based simulators (ABSs), which, joining with machine learning, simulate the consequences of self-care mindfulness programs on emotions and heart rate variability; ABSs send these repercussions to an application, guiding patients in various exercises. Following the trial of this approach, patients demonstrated their positive attitudes toward it in terms of usefulness and ease of use. A summary of the latest research on the context of telemedicine services is shown in Table 1. 

In line with earlier studies, we used the S-O-R framework to investigate the internal mechanisms between external drivers and behavioral intentions for telemedicine services. Since telemedicine is a technology-integrated platform, its nature, amenities, and functionality are essential for assessing its quality and safety [32]. Thus, the platform infrastructure and characteristics function as the stimuli that kindle the users’ internal motivations. In the current study, the stimulus is a package of features and benefits (i.e., functionality, information quality, performance expectancy, effort expectancy, facilitating condition, price value, and contamination avoidance) generated by the telemedicine platforms. In contrast, the organism is the inner experience received from telemedicine, which leads to the CUI.

**Figure 1:**
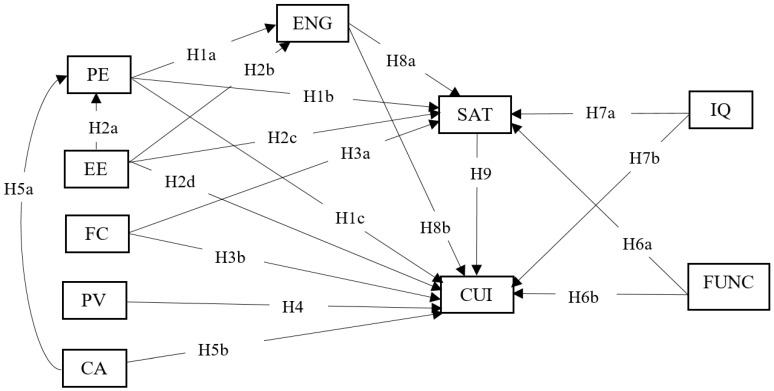
Hypothesized research model. **Mediating paths:** PE→ENG→CUI (*H10a*), EE→ENG→CUI (*H10b*), PE→SAT→CUI (*H11a*), EE→SAT→CUI (*H11b*), and FC→SAT→CUI (*H11c*). **Notes:** performance expectancy = PE, effort expectancy = EE, facilitating condition = FC, price value = PV, contamination avoidance = CA, engagement = ENG, functionality = FUNC, information quality = IQ, satisfaction = SAT, and continuous usage intention = CUI.

The organism elements we investigated in the current research are the users’ engagement and satisfaction. Our study ideates engagement as the degree to which a user believes telemedicine is an attractive, customized, responsive, and attention- and curiosity-triggering platform. In telemedicine, satisfaction is conceptualized as the users’ favorable feelings about the telemedicine services. Therefore, the current research regards engagement and satisfaction as the cognitive and affective states, respectively. We consider the CUI of telemedicine as the approach behavior, which constitutes the response variable in the S-O-R model (see Figure 1).

## 3. Hypotheses Development

### 3.1. Performance Expectancy

Performance expectancy is conceptualized as how users perceive that information technology (IT) will boost their specific performances [47]. It indicates the consumers’ expectations of improving their health quality and solving healthcare-related problems. The literature reveals that performance expectancy generates two important organismic states: engagement and satisfaction. For example, when examining the specific technology contexts, such as social networking and m-commerce apps, McLean [19] revealed that enhanced performance, impression, enjoyment, efficiency, and effectiveness from using these apps increase engagement. Similarly, when the users consider telemedicine successful in improving their health conditions, they are more likely to be satisfied with them [25,42,48].

Performance expectancy is also important to understand technology users’ post-adoption behaviors (e.g., continuance usage intention). Although a recent study by Arfi et al. [49] in the field of IoT in healthcare found no significant influence of performance expectancy on usage intentions, most recent studies, in terms of mHealth adoption [28,50,51], mobile nursing application [52], telemedicine apps [43,45,53,54], online medical records [55], and online hospitals [56], proved that performance expectancy had a significant effect on usage intention. Baudier et al. [11] and Molfenter et al. [46] also emphasized the substantial effects of PE on accepting teleconsultation solutions to avoid the risk of spread-out disease during the COVID-19 pandemic. Therefore, our study proposes the following hypothesis:

**Hypothesis** **1** **(H1).** *Performance expectancy positively influences engagement* **(H1a)** *, satisfaction* **(H1b)** *, and the CUI* **(H1c)** *of telemedicine.*

### 3.2. Effort Expectancy

Effort expectancy is conceived as the extent of ease experienced by an information system (IS) user [47]. In this study, effort expectancy is defined as perceived simplicity and convenience in using telemedicine platforms [54]. In recent studies, it has been viewed as one of the telemedicine stimuli, contributing to its perceived benefits. For instance, Octavius and Antonio [51] argued that effort expectancy significantly affects performance expectancy, signifying that the lesser the efforts invested by the user, the greater the possibility of achieving superior performance from telemedicine. Moreover, effort expectancy is an antecedent variable of engagement and satisfaction reported as in the IT literature. Previous research on m-commerce [19] and m-BI [20] demonstrated that the more the consumers perceive these technologies as straightforward and flexible, the more these will quicken their attention, interest, and curiosity. Effort expectancy enhances consumer satisfaction, as evidenced in the telemedicine literature [42,48].

Furthermore, Venkatesh et al. [30] found a significant effect of effort expectancy on the behavioral intention of using IS. Particularly, simplicity regarding user interface engineering and positioning systems of telemedicine increases usage intentions [28]. Yamin et al. [54] and Molfenter et al. [46] also established a positive link between effort expectancy and intentions to use telemedicine for health care services. Therefore, we propose the following set of hypothesis:

**Hypothesis** **2** **(H2).** *Effort expectancy has a positive influence on performance expectancy* **(H2a)** *, engagement* **(H2b)** *, satisfaction* **(H2c)** *, and the CUI* **(H2d)** *of telemedicine.*

### 3.3. Facilitating Condition

The facilitating condition is the extent to which an IS user conceives that adequate technical infrastructure is available to help them use a system [47]. Our study defines the facilitating condition as how telemedicine infrastructure, resources, and facilities support a user to use the system. Related literature embedded within the UTAUT model evinced that enabling conditions, such as users’ technical knowledge, system infrastructure, and technical support, significantly improve their satisfaction with telemedicine systems [25]. In addition, many studies in the field of telemedicine have shown that it directly influences the desire to use telemedicine services [28,51,57]. Thus, we formulate the following hypothesis:

**Hypothesis** **3** **(H3).** *Facilitating condition positively influences satisfaction***(H3a)***and telemedicine’s CUI***(H3b)**.

### 3.4. Price Value

The price value is defined as the ratio between quality and price [58]. Our study conceptualizes price value as the perceived difference between the utilities derived from telemedicine and the monetary efforts sacrificed to avail the benefits [28]. This perception of more benefits over economic cost is positive price value, which allures users’ interest in using technology [30].

Theuri [59] also mentioned that online platforms promise low-price services due to reducing the overall service cost and pushing the users to use the system. Thus, cost–benefit consideration is essential in making telemedicine services cheaper than traditional hospital services. Accordingly, Alam et al. [50] emphasized the valuation of financial viability to make telemedicine affordable. Although several researchers [28,51] have hypothesized a positive link between price value and the CUI of telemedicine services, they have not proven this to be the case. However, Dwivedi et al. [60] supported this connection in the same field. Therefore, we expect the following hypothesis:

**Hypothesis** **4** **(H4).** *Price value positively influences the CUI of telemedicine.*

### 3.5. Contamination Avoidance

Contamination avoidance refers to an individual’s tendency to avoid touching certain people or places to prevent infection [11]. It is important to address contamination avoidance because the world is still combatting COVID-19, which has resulted in thousands of deaths worldwide. During this pandemic, public places, like public transport and supermarkets, are considered more vulnerable to high infections. However, people cannot go without treatment if they become ill. Given this situation, the growing use of telemedicine may appear as a possible alternative to in-person visits to the doctor’s office. So, telemedicine may be considered one of the contamination avoidance devices, possibly protecting individuals from being infected. Recently, the usefulness of contamination avoidance has been empirically tested by a few researchers, particularly in the technology domains. While exploring mobile payment and teleconsultation adoption, Herget et al. [29] and Baudier et al. [11] indicated the substantial effect of contamination avoidance on performance expectancy. Additionally, it is a significant driver of the CUI of m-payment technology [29]. Therefore, we may also put forward the following hypothesis:

**Hypothesis** **5** **(H5).** *Contamination avoidance positively influences the performance expectancy* **(H5a)** *and the CUI* **(H5b)** *of telemedicine.*

### 3.6. Functionality

Functionality, in terms of digital health, is recognized as the degree to which a digital health tool is fast, is easy to use and navigate, and has contents that are intuitive and accurate [32]. Functionality is an IS quality measurement criterion, producing more satisfaction and usage intention [61]. Specifically, when a telemedicine user perceives it as easier to learn and navigate, more well-fixed, and structured, they will feel more positive about its usefulness, efficiency, and effectiveness [26,62]. A digital health platform user, like the IoT, will also increase their future usage rate if more features are added to improve its functional quality [63]. Therefore, we present the following hypothesis:

**Hypothesis** **6** **(H6).** *Functionality positively influences satisfaction* **(H6a)** *and the CUI* **(H6b)** *of telemedicine.*

### 3.7. Information Quality

Information quality is prescribed as a criterion that weighs the IS’s success in establishing the accuracy, relevance, security, and comprehensibility of information [61]. The current research defines information quality as the degree to which telemedicine information is understandable, appropriate, orderly, reasonably accurate, and updated. It may be expected to proliferate the telemedicine users’ affective and behavioral states, including satisfaction and the CUI. Prior research confirmed that improved information quality enhances patients’ attitudes about telemedicine services [26,27]. Moreover, its usefulness encourages consumers to use the telehealth system in the future and advise others to do the same [41]. Based on these, we propose the following hypothesis:

**Hypothesis** **7** **(H7).** *Information quality positively influences satisfaction***(H7a)***and telemedicine’s CUI***(H7b)**.

### 3.8. Engagement

Engagement, defined as the actions that promote and support a patient’s active participation in healthcare [64], has received particular attention from researchers and practitioners and has been treated as a critical healthcare policy that has embedded a new model of care, delivered to improve service quality and health outcomes [18]. People tend to support technical changes (e.g., adopting new technology), but acceptance or rejection of the change will rely on their engagement levels [17]. High engagement is the key to successful technology adoption [17,65] and satisfaction [22]. Engaged patients are more likely to be involved in preventive behaviors, self-manage symptoms and treatments, affirmatively seek health information, become advisors to new patients, and promote peer support [66]. The functional effects of engagement are also found in digital healthcare. Wang et al. [22] recently reported that healthcare professionals engaged in AI technology have higher satisfaction and usage intentions. Therefore, we propose the following hypothesis:

**Hypothesis** **8** **(H8).** *Engagement positively impacts satisfaction***(H8a)***and telemedicine’s CUI***(H8b)**.

### 3.9. Satisfaction

Satisfaction is the difference between consumer expectations and actual performance [67]. It has a long-term relationship with consumer behavioral intentions, such as continued use, word-of-mouth, repeat purchase, and loyalty. This has been proven in different contexts, including telemedicine [25]. Several researchers have reported that satisfaction positively influences patients’ continuous intentions [44,48], online and face-to-face consultation intention [68], usage intention [59], and m-health emergency use [25]. Satisfaction has also been found to positively affect AI [22]. However, travel expenses negatively affect AI [26,69], thus becoming the first encouragement of behavioral intentions in telemedicine [70]. Furthermore, if telemedicine users perceive their healthcare experiences to be excellent and convenient, this translates into use intentions [25]. Henceforth, we propose the following hypothesis:

**Hypothesis** **9** **(H9).** *Satisfaction positively impacts the CUI of telemedicine.*

### 3.10. Mediating Effect of Engagement

In the technology set, engagement, the degree of attention and interest held in using technology [71], has a successful role in its adoption [17,65]. Therefore, technology researchers place considerable importance on operating studies, which discern the ways and facts to improve engagement [19]; this engagement affects consumers’ long-term behavioral outcomes, like AI usage intentions in healthcare [22] and social network usage in sales [72]. The stimulating variables in technology adoption were indicated to directly influence consumers’ behavioral intentions [29,41,54]. Thus, the mediation test for engagement is reasonable in the technology domain because, without it, we cannot understand the perplexing interplay between the study variables.

In the field of HIMS, Omuudu et al. [24] reported engagement as a significant mediator in the relationship between perceived usefulness and ease of use to innovative work behavior. Peters et al. [20] confirmed the indirect effect of m-BI characteristics, including usefulness, accessibility, flexibility, and interface attractiveness, on the usage frequency via engagement. Abou-Shouk and Soliman [23], in their gamification research, revealed a partial mediation influence of engagement on the linkage between game app adoption intention and brand awareness and loyalty. Nevertheless, the mediating trial of engagement in the relationship between telemedicine stimuli and usage intentions has been neglected in the literature. Only Wang et al. [22], having validated engagement as a mediatory mechanism between the attributes of AI adoption in healthcare and behavioral intentions, are motivators for the current study. Given the above discussions, we offer the following hypothesis:

**Hypothesis** **10** **(H10).** *Engagement mediates the link between performance expectancy and the CUI* **(H10a)** *, effort expectancy, and the CUI* **(H10b)** *of telemedicine.*

### 3.11. Mediating Effect of Satisfaction

We ratiocinate that the antecedents (i.e., performance expectancy, effort expectancy, and facilitating condition) of telemedicine adoption influence users’ behavioral intentions by improving their levels of satisfaction with it. The technology stimuli, such as performance expectancy, effort expectancy, and facilitating condition, have significant roles in telemedicine use [28,53,54]. The relevant literature also showed that the more the consumers perceive telemedicine as helpful, easy to use, and technically supportive, the more they will show positive feelings toward it [48], which will affect usage intentions [25]. Despite the need for mediation examination of satisfaction, research is scant in the telemedicine literature. As far as we know, only Zhou et al. [27] uncovered a critical mediation effect of satisfaction on the connection between effort expectancy and information quality, and telehealth usage motives, thus paving the way to verify such an important mediation test.

Over and above this, satisfaction was empirically justified as a mediating variable in other technology fields. For instance, in the automatic banking context, Rahi et al. [53] reported on the significant effects of automated banking attributes, including service quality, website design, and brand image, on adoption desires through satisfaction. In the same environment, Mohammed and Ward [73] noted the mediating role of satisfaction between service quality and the financial performance of banks. In addition, Iqbal et al. [74] acknowledged a partial mediating influence of satisfaction on the interplay between service quality and the desire to use self-service technology. These empirical insights led us to formulate the following statement:

**Hypothesis** **11** **(H11).** *Satisfaction mediates the link between performance expectancy and the CUI* **(H11a)** *, effort expectancy and the CUI* **(H11b)** *, facilitating condition, and the CUI* **(H11c)** *of telemedicine.*

## 4. Research Methodology

### 4.1. Instrument Development

This study used a questionnaire instrument from the existing literature, and to adapt to the context, we conducted a focused group study with two researchers and two healthcare practitioners. Initially, the questionnaire was developed in English and then translated to Bangla by a specialist; after verification and modification, we used both languages in the final survey. We adopted the questions from the existing literature for measuring performance expectancy, effort expectancy, facilitating condition, price value, contamination avoidance, engagement, functionality, information quality, satisfaction, and the CUI (see Appendix A Table A1). A seven-point Likert scale, where one denotes strongly disagree and seven strongly agree, was used for the responses.

### 4.2. Data Collection and Sample

**Figure 2:**
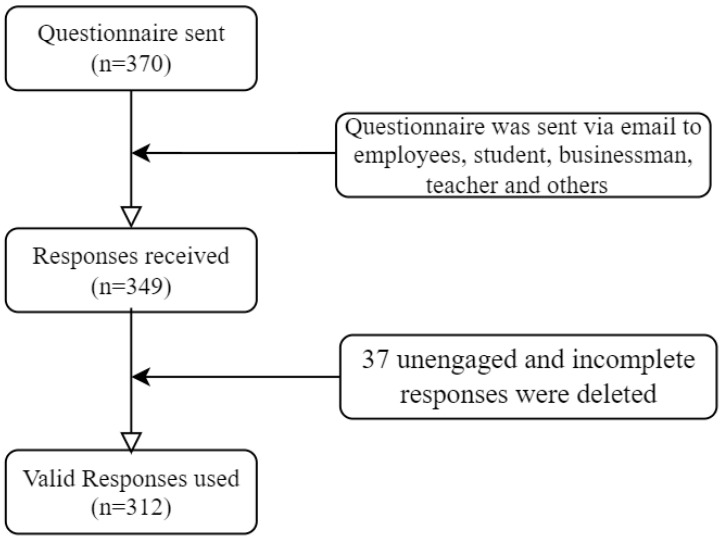
Participants’ inclusion flowchart.

Using a convenience sampling method, we conducted an online survey of patients with prior experience using telemedicine services in Bangladesh. At the beginning of the questionnaire, a summary of the study objectives was given to receive anonymous responses from the participants. To select target samples, participants were instructed to respond only if they had previous experience with telemedicine services. Before the final survey, we pretested our questionnaire with 30 samples, and necessary modifications and changes were made accordingly. We administered the final survey from 5 October 2021 to 10 November 2021. Initially, 349 samples were collected; after data cleaning (i.e., removing unengaged and incomplete responses), 312 valid samples were kept for final analysis. Figure 2 shows the recruitment flowchart. The data were analyzed using the structural equation model (SEM) technique in Amos 24.0 software. A two-step procedure, including measurement and structural models, was performed to provide better reliability, validity, and structural relationships among exogenous variables [75].

## 5. Empirical Results

### 5.1. Participants’ Demographics

Descriptive statistics of the samples are shown in Table 2, denoting that the gender distribution was somewhat skewed, with 74% males and 25% females. The average age of the participants was 26.28, and the distributions were 44% for 21–25 years, 33% for 26–30 years, and 8% for 31–35 years. Young participants dominated our sample, indicating that they might have greater access to up-to-date information and be more technologically competent. Most of the participants were students (55%) and teachers (18%). Descriptive statistics also show that 50% of the participants were urban people, and suburban and rural participants comprised 20% and 29%, respectively. Furthermore, telemedicine service was used over the past six months by our study participants once (35%), twice (25%), and many times (34%), and the average usage was 2.39 times.

### 5.2. Measurement Model Reliability and Validity

**Figure 3:**
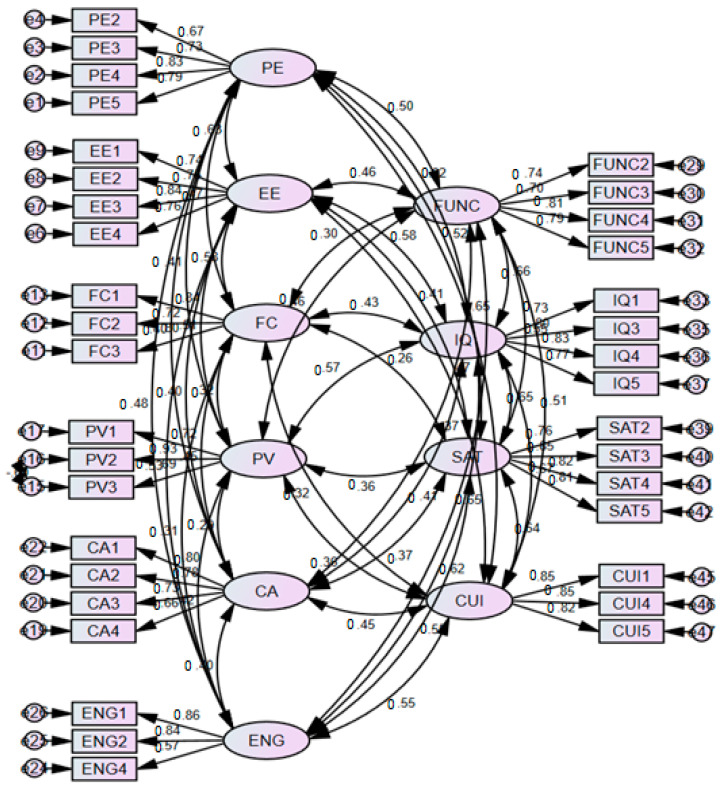
Measurement model.

The measurement model (see Figure 3) was assessed through reliability, convergent, and discriminant validity. First, to determine reliability, we examined factor loadings and Cronbach’s alpha; an alpha value >0.70 was considered satisfactory [76]. Table 3 demonstrates that all the factor loadings and Cronbach’s alpha values are greater than their respective thresholds, revealing that they are reliable for our study. In order to examine convergent validity, we reviewed and accepted composite reliability (CR) with a value >0.70 and average variance extracted (AVE) with a value >0.50, because these values were considered satisfactory [77]. Our calculated values (i.e., CR > 0.70, AVE > 0.50) show sufficient evidence for the convergent validity of the data.

Then, to measure the discriminant validity, we compared the square root of the AVE of each factor with the correlation value shared with all other constructs. If the square root of AVE was higher than the correlation value, discriminant validity was satisfied [78]. The results in Table 4 show that our data successfully satisfied the discriminant validity criteria.

### 5.3. Method Bias Test

After that, we conducted a method bias test; since the data were collected at once from a single source, there was the possibility of method bias. In order to deal with method bias, we conducted a single factor test according to the principles of Podsakoff et al. [79]. The principal component factor analysis with an un-rotation matrix shows that the first factor explained 34% of the variance, and several factors had eigenvalues >1. In addition, we examined the variance inflation factor (VIF), which should be lower than 3.3 [80]. The VIF (see Table 4) values were between 1.32 and 2.34 in our study and were within their suggested thresholds. This reveals that there is no severe concern of method bias in our data. These VIF values also confirm no multicollinearity issues in our data, as values are lower than 3.

### 5.4. Model Fit Test

Furthermore, we assessed some popular model fit indices to validate our model’s fitness. The values of all the standard fit indices are within their critical limits, such as chi-square to degrees of freedom (CMIN/df = 1.854), comparative fit index (CFI = 0.925), goodness-of-fit index (GFI = 0.853), adjusted GFI (AGFI = 0.822), normalized fit index (NFI = 0.853), Tucker-Lewis index (TLI = 0.914), incremental fit index (IFI = 0.926), and root mean square error of approximation (RMSEA = 0.052), which indicates that the research model has a good fit as a whole [76,81].

### 5.5. Structural Model Analysis

**Figure 4:**
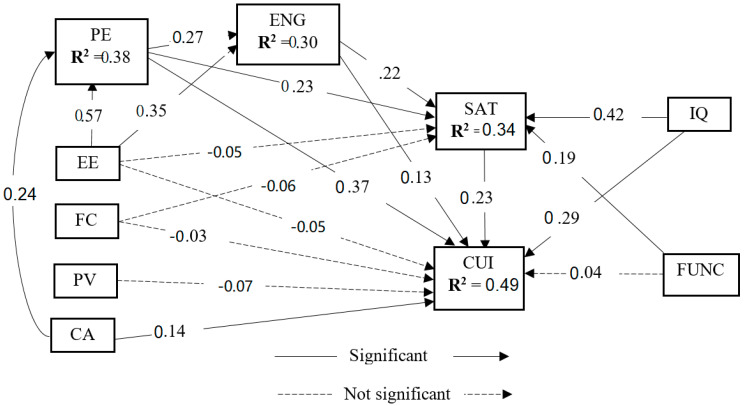
Structural model. **Note:** All mediating hypotheses, i.e., *H10a* (PE→ENG→CUI), *H10b* (EE→ENG→CUI), *H11a* (PE→SAT→CUI), and *H11b* (EE→SAT→CUI), except *H11c* (FC→SAT→CUI), are significant.

The results of the structural model are shown in Figure 4. Fit indices for the structural model also met the recommended values: CMIN/df = 2.861, CFI = 0.830, GFI = 0.756, AGFI = 0.717, NFI = 0.762, TLI = 0.813, IFI = 0.831, and RMSEA = 0.077 (see Table 5). The variance explained by our structural model is 38% in performance expectancy, 30% in engagement, 34% in satisfaction, and 49% in the CUI, which reveal that our model has sufficiently validated the proposed objectives.

Path coefficient (*β*), *t*-value < 0.001, and *p*-value < 0.05 are considered to assess the proposed hypothetical relationships. Detailed hypothesis test results are shown in Table 6. The results show that H1a-c, H2a-b, H5a-b, H6a, H7a-b, H8a-b, and H9 are accepted, but H2c-d, H3a-b, H4, and H6b are rejected. Performance expectancy strongly correlates with the CUI of telemedicine services, and information quality strongly correlates with satisfaction.

As a post hoc analysis, we conducted mediation analysis using bootstrapping according to the Baron and Kenny [82] principles. Results show that engagement and satisfaction significantly mediate between performance expectancy to the CUI and effort expectancy to the CUI.

## 6. Discussion

Telemedicine is rapidly changing the face of healthcare delivery. There are dozens of healthcare applications on the internet to improve people’s access to healthcare. However, as indicated in the literature, the telemedicine stakeholders face the challenges of engaging users engaged over the long term. Thus, this research intends to investigate the impacts of perceived stimuli on the CUI of telemedicine services along with the mediation influences of engagement and satisfaction. The study developed 24 hypotheses; out of these, 17 hypotheses were supported, and 7 were rejected. Firstly, H1a, H1b, and H1c proposed that performance expectancy significantly influences engagement, satisfaction, and the CUI of telemedicine. Our analyses confirmed these hypotheses, which is identical to the results of past studies [48,54]. The results also supported H2a and H2b, which found that effort expectancy substantially and positively influenced performance expectancy and engagement. These outcomes are consistent with those of earlier research [19,51], signifying that telemedicine providers should prioritize adding more convenient and easy-to-use features to the system to improve its performance and consumer engagement. However, the results could not confirm H2c and H2d, which investigated if effort expectancy influences satisfaction and the CUI; these findings are not similar to past research [28,48]. Furthermore, we failed to verify H3a and H3b, investigating whether facilitating condition increases user satisfaction and the CUI, which disagrees with the literature [25]. In addition, H4, offering a positive influence of price value on the CUI, was not supported and thus contrasted with the prior studies [60].

We hypothesized that contamination avoidance affects performance expectancy (H5a) and the CUI (H5b), which the present study proved, as did recent studies [11,29], meaning that contamination avoidance is a significant attribute of an IS and accelerates its performance and usage intentions, especially during a pandemic situation like COVID-19. In addition, H6a inquired into and corroborated the effect of the functionality on satisfaction, which agrees with the outcome of previous studies [26,62]. The influence of functionality on the CUI (H6b) was not affirmed, thus contradicting past research findings [64]. The present study supported H7a and H7b, which stated that information quality has a critical effect on satisfaction and the CUI; these results are consistent with those of preceding studies [26,27,41]. Moreover, the impacts of engagement on satisfaction (H8a) and the CUI (H8b) were recognized by both the current and former studies [22]. Telemedicine literature [48,68] revealed that satisfaction is a significant antecedent to the CUI; we also found the same result, that is, H9 was also confirmed.

This study also investigated and demonstrated engagement as a mediator between performance expectancy and the CUI (H10a) and between effort expectancy and the CUI (H10b); past researchers [23,24] also indicated similar results, but in general technology domains like m-BI and gaming apps. Finally, the effects of performance expectancy and effort expectancy on the CUI mediated by satisfaction (i.e., H11a and H11b) were also advocated by the current study and past analyses performed by Zhou et al. [27], Rahi et al. [53], and Iqbal et al. [74]. On the other hand, H11c stated that satisfaction mediates between facilitating condition and CUI, but our analyses do not support this hypothesis.

## 7. Contributions of the Study

### 7.1. Theoretical Contributions

Our study is mainly based on the S-O-R framework coupled with the UTAUT2, A-MARS, and ECM models, which have not been combined previously in the telemedicine literature. In addition, conjoining the contamination avoidance, information quality, and functionality with the UTAUT2 constructs stretches the scope of the UTAUT2 model, particularly in telemedicine services. The findings of this study enrich the existing literature by introducing new hypotheses embedded in the S-O-R and UTAUT frameworks that have not yet been tested in telemedicine. For instance, presenting the direct effects of performance expectancy and effort expectancy on engagement (i.e., performance expectancy→engagement, and effort expectancy→engagement) expands the telemedicine literature. In addition, the functional consequences of engagement were tested in the domain of automating telemedicine but not synchronous telemedicine services. Thus, the current research contributes to the literature by disclosing that engagement gives rise to positive emotional and behavioral outcomes, such as satisfaction and the CUI (engagement→satisfaction and engagement→CUI).

Moreover, prior research has shown the direct effects of performance expectancy and effort expectancy on usage intention. In contrast, our findings become the pathfinder for proving that such influences are significantly mediated by engagement (i.e., performance expectancy→engagement→CUI and effort expectancy→engagement→CUI)**.** Additionally, the support for the mediation role of satisfaction in between performance expectancy and CUI (i.e., performance expectancy→satisfaction→CUI) gives novelty to the current state of knowledge regarding telemedicine adoption. Though H11c (facilitating condition→satisfaction→CUI) seems likely in telemedicine, the analyses could not report it as significant. However, we believe it will leave a blueprint for future researchers to construct a model with the variables best compatible with telemedicine research. Finally, the significant influence of contamination avoidance on CUI, supported by our results, captures a new position in telemedicine research.

### 7.2. Practical Contributions

The results of this research can be used by concerned stakeholders, such as telemedicine service providers, developers, marketers, managers, healthcare institutions, medical policymakers, and the government, to formulate policies and blueprints for implementing and promoting the CUI of telemedicine among the population in a developing economy like Bangladesh. Focusing on the central findings, the power of performance expectancy, contamination avoidance, information quality, engagement, and satisfaction should be weighed by the concerned stakeholders in sustaining the usage intention. Notably, more importance should be given to the influences of performance expectancy, information quality, and satisfaction, because they have the most potent predicting power over the CUI of telemedicine. In addition, following the crucial roles of effort expectancy and contamination avoidance in performance expectancy, the telemedicine developers should design systems to make it easier and more feasible to avoid infectious diseases like COVID-19 to enrich the perceived usefulness of telemedicine. Given the significant outcomes of the mediation tests, managers should attach importance to engagement and satisfaction to ensure that the users remain persistent in using telemedicine in the future. They also should consider the roles of performance expectancy and effort expectancy in improving user engagement levels and performance expectancy, functionality, information quality, and engagement in increasing user satisfaction. The role of effort expectancy in CUI played via both engagement and satisfaction should be noted, as it has the highest indirect effect on the CUI (see Table 7). According to these results, telemedicine designers and developers should incorporate and upgrade these attributes into the system to make it more user-friendly. However, given the non-significant roles of price value and facilitating condition, managers are recommended not to decrease the current prices or increase the available resources, such as manuals, when using telemedicine to augment the CUI.

Furthermore, the government and marketers should promote telemedicine services among the country’s citizens as an effective mechanism for minimizing the spread of contagious ailments like COVID-19. The country’s health institutions, including hospitals, clinics, and pharmaceutical companies, should build an alliance with the telemedicine service providers to popularize this disruptive platform among the public. Most importantly, telemedicine providers should spur their consumers to use different clinical-grade wearables, such as smart belts, watches, rings, and clothes. These devices enable the patients to independently perform and share their primary health check-ups, including blood pressure, heart rate, and body temperature, to the concerned doctors, which might make these platforms more engaging for the users. Consequently, the integrated efforts from all concerned could promote a sustainable ecosystem for telemedicine services in a context where ICT is stagnant, like Bangladesh.

## 8. Conclusions, Limitations, and Blueprints for Future Research

The present study reveals that telemedicine consumers’ usage intentions are directly influenced by performance expectancy, information quality, contamination avoidance, engagement, and satisfaction. Moreover, the impacts of performance and effort expectancies on the continuous usage intention are facilitated via user engagement and satisfaction, which are novel in the telemedicine literature as embedded within the S-O-R and UTAUT paradigms. In addition, performance and effort expectancies are crucial in building user engagement; performance expectancy, functionality, information quality, and engagement are the significant antecedents to amplifying user satisfaction. The telemedicine facilitators, policymakers, promoters, and the federal authorities could apply these outcomes in envisioning and executing strategies for increasing the continuous usage intentions of telemedicine in a developing context like Bangladesh.

Though this research has reported various significant conclusions regarding telemedicine adoption, several limitations encircle the study, which might affect the perspectives of this study. Firstly, the survey was conducted among telemedicine users during the COVID-19 pandemic. However, the adoption patterns of telemedicine might be varied beyond COVID-19; therefore, longitudinal research is required to depict the actual scenario of usage intention of telemedicine services. Secondly, this study performs the mediation tests in terms of performance expectancy, effort expectancy, and facilitating condition only; however, other variables like functionality and information quality might affect future desires to adopt telemedicine through engagement and satisfaction. Thirdly, this study has limited itself to examining the roles of only two affective components, i.e., engagement and satisfaction; however, other individual contributory factors like personal innovativeness could influence the CUI of telemedicine. Fourthly, this study ignores the moderation tests, unlike the UTAUT model; hence, future researchers should perform moderation examinations of respondents’ age, gender, place of residence, and frequency of use on the relationships offered in the expanded model to obtain a better picture. Fifthly, the current analyses could not report the statistically significant effects of price value and facilitating condition, which are rarely reported in past studies in developing economies; other researchers should investigate further the roles of these variables in the telemedicine domain. Moreover, the majority of the respondents of this study are students and teachers, meaning that other clusters of society were overlooked, such as medical professionals, business people, private and government job holders, the unemployed, and others. Finally, the current model can be tested in similar contexts like Pakistan and India.

## Figures and Tables

**Table 1:** Summary of the latest research on the context of telemedicine services in both developed and developing economies.

Authors	Main Objective	Significant Findings/Hypotheses	Limitations	Proposed Solution
Bamufleh et al. [41]	To examine Saudi Arabians’ adoption intentions of e-government health applications during COVID-19.	ease of use→usefulness usefulness→ behavioral intention attitude→behavioral intention information quality→usefulness trust→attitude social influence→behavioral intention facilitating condition→behavioral intention	These authors did not perform any mediation test of organismic variables like attitude.	Our study explores if cognitive and affective variables like engagement and satisfaction mediate the paths between external stimuli and usage intention of telemedicine.
An et al. [42]	To explore the factors motivating the South Korean citizens to accept telemedicine services during the pandemic.	increased accessibility→usefulness enhanced care→usefulness ease of use→usefulness usefulness→attitude ease of use→attitude privacy and discomfort→attitude (negative relation) attitude→intention to use		
Alam et al. [28]	To examine the factors of behavioral intention and actual usage behavior of Bangladeshi mobile health users and explore the impact of actual usage behavior on their mental well-being.	performance expectancy→behavioral intention effort expectancy→behavioral intention social influence→behavioral intention facilitating condition→behavioral intention habit→behavioral intention health consciousness→behavioral intention facilitating condition→actual usage behavior health consciousness→actual usage behavior behavioral intention→actual usage behavior self-quarantine→actual usage behavior actual usage behavior→mental well-being		
Baudier et al. [11]	To investigate the usage intention of telemedicine services among end-users in Italy, France, the UK, and China.	habit→intention to use performance expectancy→intention to use risk→intention to use (negatively related) self-efficacy→effort expectancy personal innovativeness→effort expectancy availability→performance expectancy contamination avoidance→performance expectancy		
Ouimet et al. [43]	To investigate Canadian patients’ continuous usage intention of telemedicine services.	usefulness→continuance usage intention quality→continuance usage intention quality→usefulness quality→trust expectation confirmation→quality expectation confirmation→usefulness	These authors did not use any theoretical framework to develop their research model. They also examined the consumers’ usage intention with the inclusion of a handful of variables, which might narrow the perspective of the study.	Our research is based on the S-O-R framework. It examines the context of telemedicine by adding seven stimuli, two organismic factors, and one response variable, which might broaden our study’s perspective.
Luo et al. [44]	To investigate the drivers of continuous usage intentions of telemedicine apps among Chinese residents.	vulnerability→self-efficacy vulnerability→response efficacy self-efficacy→attitude response efficacy→attitude self-efficacy→continued intention response efficacy→continued intention direct network externalities→attitude indirect network externalities→attitude indirect network externalities→continued intention attitude→continued intention self-efficacy→attitude→continued intention response efficacy→attitude→continued intention direct network externalities→attitude→continued intention indirect network externalities→attitude→continued intention	This study mainly emphasized the psychological factors of using telehealth apps.	The present model offers a possible solution to this limitation by adding telemedicine interface, attributes, and performance-related variables, such as performance and effort expectancies, functionality, information quality, etc.
Serrano et al. [45]	To examine the factors that influence telemedicine acceptance among adults in Brazil and test the moderation effects of disease complexity and the digital age on these relationships.	performance expectancy→intention to use security and reliability→intention to use security and reliability→performance expectancy	These studies did not use any psychological variables to examine the context of telemedicine.	We incorporate one cognitive variable (i.e., engagement) and one affective variable (i.e., satisfaction) into our model.
Molfenter et al. [46]	To investigate healthcare providers’ usage intentions of synchronous and asynchronous telemedicine services in the USA.	ease of use→future intention to use ease of use→usefulness usefulness→future intention to use ease of use→usefulness→future intention to use		

**Table 2:** Demographic statistics (*n* = 312).

Characteristics	Frequency	Percentage
Gender		
Female	80	25.64
Male	232	74.35
Age group (mean = 26.28, std. deviation = 5.53)		
Below 20	15	4.80
21–25	138	44.23
26–30	105	33.65
31–35	26	8.33
36–40	18	5.76
Above 40	10	3.20
Occupation		
Private employee	19	6.1
Government employee	19	6.1
Student	173	55.4
Business	2	0.6
Teacher	58	18.6
Unemployed	17	5.4
Other	24	7.7
Place of residence		
Urban	156	50.0
Suburban	64	20.5
Rural	92	29.48
How often did you use telemedicine services over the last six months? (mean = 2.39, std. deviation = 1.31)		
Once	111	35.6
Twice	78	25.0
Three times	16	5.1
Many times	107	34.3

**Table 3:** Reliability and validity.

Constructs		Estimate	CR	AVE	MSV	MaxR(H)	Alpha Value
PE	PE5	0.788	0.841	0.572	0.424	0.852	0.840
	PE4	0.829					
	PE3	0.725					
	PE2	0.673					
EE	EE4	0.756	0.855	0.596	0.396	0.861	0.851
	EE3	0.837					
	EE2	0.749					
	EE1	0.742					
FC	FC3	0.801	0.831	0.622	0.340	0.841	0.824
	FC2	0.720					
	FC1	0.841					
PV	PV3	0.686	0.829	0.622	0.324	0.896	0.780
	PV2	0.932					
	PV1	0.725					
CA	CA4	0.663	0.833	0.556	0.203	0.840	0.828
	CA3	0.732					
	CA2	0.782					
	CA1	0.798					
ENG	ENG4	0.570	0.808	0.591	0.421	0.852	0.782
	ENG2	0.841					
	ENG1	0.860					
FUNC	FUNC2	0.744	0.846	0.580	0.436	0.852	0.842
	FUNC3	0.697					
	FUNC4	0.806					
	FUNC5	0.794					
IQ	IQ1	0.726	0.862	0.611	0.449	0.867	0.848
	IQ3	0.802					
	IQ4	0.829					
	IQ5	0.765					
SAT	SAT2	0.765	0.884	0.657	0.420	0.887	0.884
	SAT3	0.845					
	SAT4	0.821					
	SAT5	0.809					
CUI	CUI1	0.848	0.879	0.708	0.449	0.880	0.877
	CUI4	0.855					
	CUI5	0.821					

**Table 4:** Discriminant validity.

	SAT	PE	EE	FC	PV	CA	ENG	FUNC	IQ	CUI	VIF
SAT	**0.811**										1.67
PE	0.515	**0.756**									1.74
EE	0.412	0.629	**0.772**								1.93
FC	0.255	0.470	0.583	**0.789**							1.48
PV	0.363	0.408	0.509	0.324	**0.788**						1.49
CA	0.370	0.403	0.399	0.451	0.292	**0.746**					1.32
ENG	0.547	0.482	0.526	0.306	0.421	0.398	**0.769**				1.87
FUNC	0.550	0.499	0.462	0.305	0.456	0.367	0.649	**0.761**			1.80
IQ	0.648	0.620	0.582	0.428	0.569	0.408	0.618	0.660	**0.781**		2.34
CUI	0.636	0.651	0.466	0.324	0.357	0.446	0.553	0.514	0.670	**0.841**	

**Note:** Bold diagonal values are the square roots of AVEs.

**Table 5:** Model fit results.

Indices	Recommended Value	The Obtained Value Measurement Model	The Obtained Value Structural Model
CMIN/df	<3	1.854	2.861
CFI	≥0.90	0.925	0.830
GFI	≥0.80	0.853	0.756
AGFI	≥0.80	0.822	0.717
NFI	≥0.90	0.853	0.762
TLI	≥0.90	0.914	0.813
IFI	≥0.90	0.926	0.831
RMSEA	≤0.08	0.052	0.077

**Source:** Data analysis results.

**Table 6:** Hypothesis results.

Hypothesized Paths			Estimate	SE.	CR.	P	Decision
**H1a:** PE	--->	ENG	0.271	0.063	3.325	***	Accept
**H1b:** PE	--->	SAT	0.227	0.073	2.855	***	Accept
**H1c:** PE	--->	CUI	0.366	0.079	4.479	***	Accept
**H2a:** EE	--->	PE	0.572	0.060	8.322	***	Accept
**H2b:** EE	--->	ENG	0.345	0.057	4.096	***	Accept
**H2c:** EE	--->	SAT	−0.052	0.063	−0.664	n.s.	Reject
**H2d:** EE	--->	CUI	−0.052	0.063	−0.692	n.s.	Reject
**H3a:** FC	--->	SAT	−0.056	0.055	−0.979	n.s.	Reject
**H3b:** FC	--->	CUI	−0.035	0.053	−0.659	n.s.	Reject
**H4:** PV	--->	CUI	−0.066	0.057	−1.306	n.s.	Reject
**H5a:** CA	--->	PE	0.237	0.076	3.924	***	Accept
**H5b:** CA	--->	CUI	0.138	0.070	2.389	**	Accept
**H6a:** FUNC	--->	SAT	0.187	0.050	3.169	***	Accept
**H6b:** FUNC	--->	CUI	0.037	0.049	0.675	n.s.	Reject
**H7a:** IQ	--->	SAT	0.422	0.069	6.348	***	Accept
**H7b:** IQ	--->	CUI	0.293	0.071	4.543	***	Accept
**H8a:** ENG	--->	SAT	0.217	0.089	2.877	***	Accept
**H8b:** ENG	--->	CUI	0.129	0.086	1.857	**	Accept
**H9:** SAT	--->	CUI	0.234	0.075	3.307	***	Accept
**Variance explained**			**R squared**				
PE			0.383				
ENG			0.300				
SAT			0.339				
CUI			0.493				

**Note:** *** *p* < 0.001, ** *p* < 0.05, and n.s. = not significant.

**Table 7:** Bootstrapping results.

Path	Indirect Effect	Lower Bound	Upper Bound	*p*-Value	Decision
**H10a:** PE-ENG-CUI	0.080	0.011	0.208	***	Mediated
**H10b:** EE-ENG-CUI	0.489	0.337	0.701	***	Mediated
**H11a:** PE-SAT-CUI	0.175	0.082	0.335	***	Mediated
**H11b:** EE-SAT-CUI	0.433	0.286	0.604	***	Mediated
**H11c:** FC-SAT-CUI	−0.001	−0.084	0.077	n.s.	Not mediated

**Note:** *** *p* < 0.001 and n.s. = not significant.

## Data Availability

Data are available upon special request from the corresponding author.

## Appendix A

**Table A1:** Measurement Instruments.

Constructs (Sources)	Items	Mean	Std. Dev.	Statements
Performance Expectancy (PE) [11,30]	PE2	5.92	1.190	Telemedicine would allow me to access healthcare services faster.
	PE3	5.54	1.213	Telemedicine services improve my healthcare efficiency.
	PE4	5.41	1.262	Telemedicine services increase my capability to manage my health more quickly.
	PE5	5.62	1.178	Telemedicine would increase my chances of meeting my healthcare needs.
Effort Expectancy (EE) [30]	EE1	5.75	1.238	Learning to use telemedicine is effortless for me.
	EE2	5.29	1.461	My interaction with telemedicine is understandable.
	EE3	5.60	1.336	I find telemedicine platforms easy to use.
	EE4	5.69	1.379	It is simple to be skillful at using telemedicine services.
Facilitating Conditions (FC) [47]	FC1	6.22	1.184	I have the essential resources to use telemedicine.
	FC2	5.89	1.276	I have the necessary knowledge to use telemedicine.
	FC3	6.07	1.052	The telemedicine service I have used can run on both computer and mobile phone without modifications.
Price Value (PV) [30]	PV1	5.00	1.583	The fees or prices for telemedicine services (e.g., doctor’s fees) are reasonable.
	PV2	5.12	1.574	The fees for telemedicine services are affordable.
	PV3	4.83	1.630	Telemedicine services are good value for the money.
Contamination Avoidance (CA) [11]	CA1	5.81	1.293	Telemedicine allows me to avoid a physical visit to the doctor’s office.
	CA2	6.15	.977	Telemedicine allows me to avoid physical contact with other patients.
	CA3	5.96	1.140	Telemedicine allows me to avoid physical contact with the doctor.
	CA4	6.24	1.086	Telemedicine allows me to avoid touching infected objects (e.g., door handles, chairs)
Functionality (FUNC) [32]	FUNC2	5.34	1.337	The sign-up and sign-in processes of the telemedicine service are quick and simple.
	FUNC3	5.12	1.403	The telemedicine service I have used has relevant help buttons/FAQs.
	FUNC4	5.40	1.215	The menu labels, icons, and instructions of the telemedicine service I have used are straightforward.
	FUNC5	5.58	1.148	The telemedicine service allows me to navigate or move from one section to another easily.
Information Quality (IQ) [32]	IQ1	5.48	1.167	It is effortless to find and understand information on the telemedicine platform.
	IQ3	5.58	1.114	The information available on telemedicine platforms is orderly and easy to read.
	IQ4	5.49	1.137	Information provided by the telemedicine platform is correct and relevant.
	IQ5	5.50	1.162	Information provided by the telemedicine platform is timely and updated.
Engagement (ENG) [32]	ENG1	5.63	1.254	Telemedicine technology is engaging.
	ENG2	5.51	1.237	Telemedicine technology is interesting.
	ENG4	5.63	1.316	The telemedicine platform is responsive and holds my attention.
Satisfaction (SAT) [47,67,83]	SAT2	5.44	1.217	I am pleased with my experience with telemedicine service.
	SAT3	5.34	1.313	The experience of telemedicine service is exactly what I needed.
	SAT4	5.44	1.276	I think I did the right thing when I decided to use the telemedicine service.
	SAT5	5.60	1.248	I like using telemedicine services.
Continuous Usage Intention (CUI) [31,48]	CUI1	5.67	1.163	I intend to continue using telemedicine services.
	CUI4	5.68	1.149	I will recommend others to use telemedicine platforms.
	CUI5	5.39	1.291	I plan to continue to use telemedicine services frequently.
